# Crystal structure of aqua­{μ-*N*-[3-(di­methyl­amino)­prop­yl]-*N*′-2-(oxidophen­yl)oxamidato}(1,10-phen­anthroline-5,6-dione)dicopper(II) perchlorate hemihydrate

**DOI:** 10.1107/S2056989015009391

**Published:** 2015-05-23

**Authors:** Xin Zhang, Yan-Tuan Li, Zhi-Yong Wu

**Affiliations:** a Key Laboratory of Marine Drugs, Ministry of Education of China, School of Medicine and Pharmacy, Ocean University of China, Qingdao 266003, People’s Republic of China

**Keywords:** crystal structure, binuclear copper(II) complex, oxamide complex, 1,10-phenanthroline-5,6-dione, hydrogen bonding, π–π stacking

## Abstract

The title compound, [Cu_2_(C_13_H_16_N_3_O_3_)(C_12_H_6_N_2_O_2_)(H_2_O)]ClO_4_·0.5H_2_O, consists of a *cis*-oxamide-bridged binuclear Cu^II^ complex cation, a perchlorate anion and half a solvent water mol­ecule. One Cu^II^ cation is *N*,*N*′,*N*",*O*-chelated by an *N*-[3-(di­methyl­amino)­prop­yl]-*N*′-(2-hy­droxy­phen­yl)oxamide trianion in a distorted square-planar geometry, whereas the other Cu^II^ cation is *O*,*O*′-chelated by the oxamide moiety of the anion and *N*,*N*′-chelated by a 1,10-phenanthroline-5,6-dione mol­ecule, and a water mol­ecule further coordinates the second Cu^II^ cation, completing a distorted square-pyramidal coordination geometry. In the crystal, classical O—H⋯O hydrogen bonds, weak C—H⋯O hydrogen-bonding inter­actions and π–π stacking inter­actions link the complex cations, anions and solvent water mol­ecules into a three-dimensional supra­molecular architecture. In the crystal, the di­methyl­amino­propyl unit of the oxamide anion is disordered over two positions with an occupancy ratio of 0.561 (11):0.439 (11); the solvent water mol­ecule is also disordered over two positions, the occupancy ratio being 0.207 (10):0.293 (10).

## Abstract 2

The *N*-[3-(di­methyl­amino)­prop­yl]-*N*′-(2-hy­droxy­phen­yl)oxamide trianion bridges two Cu^II^ cations to form the binuclear complex, in which the Cu^II^ cations have distorted square-planar and square-pyramidal coordination geometries.

## Chemical context   

It is known that oxamide ligands could be good candidates for forming polynuclear complexes because of their versatile coordinating abilities (Ojima & Nonoyama, 1988 ▸; Ruiz *et al.*, 1999 ▸). Therefore, many oxamide complexes and their properties have been investigated extensively (Messori *et al.*, 2003 ▸; Wang *et al.*, 2013 ▸; Li *et al.*, 2011 ▸).
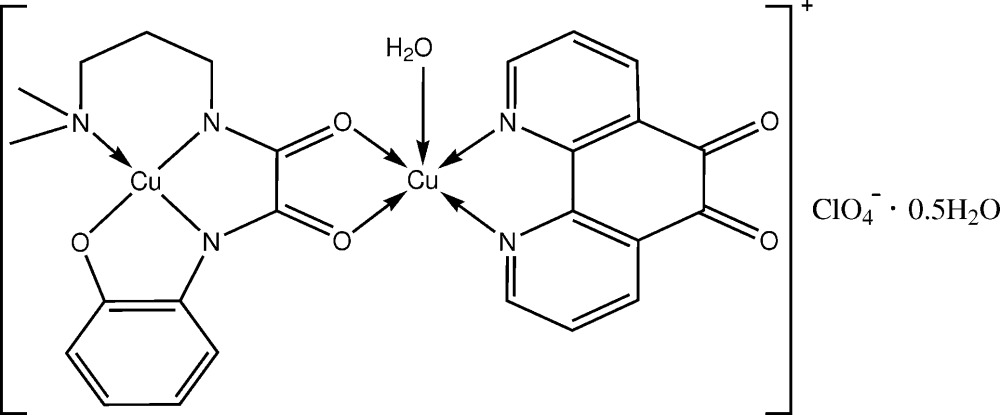


 1,10-Phenanthroline-5,6-dione (Phdo) is a multifaceted ligand since the structure and electronic properties thereof incorporate the features of the di­imine and quinone functionalities (Girgis *et al.*, 1975 ▸; Calderazzo *et al.*, 2002 ▸). Consequently, as part of our systematic study of asymmetrical bis-substituent oxamide complexes and the influence of structures on the DNA-binding properties thereof (Li *et al.*, 2012 ▸; Zhang *et al.*, 2013 ▸), we selected *N*-[3-(di­methyl­amino)­prop­yl]-*N*′-2-(oxidophen­yl)oxamide (H_3_Dmapox) as a bridg­ing ligand and Phdo as a terminal ligand to synthesize the title binuclear copper(II) complex, [Cu_2_(Dmapox)(Phdo)H_2_O]^+^ClO_4_^−^·0.5H_2_O. Its crystal structure and supra­molecular structure are reported here.

## Structural commentary   

**Figure 1:**
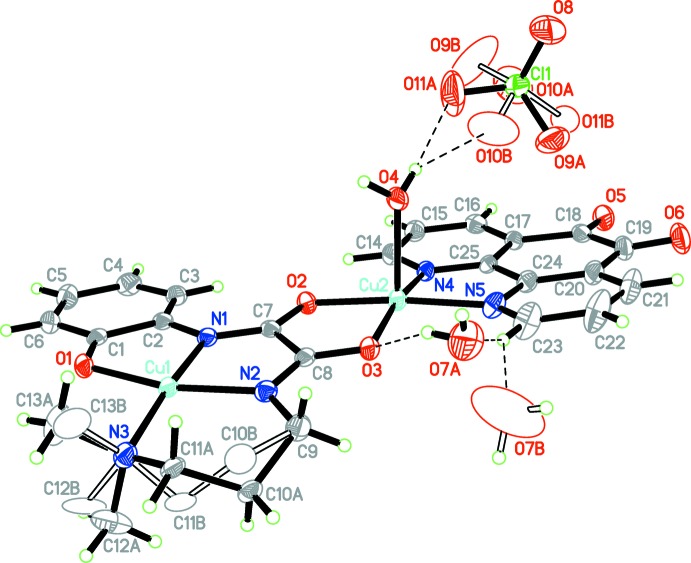
The mol­ecular structure of the title compound with displacement ellipsoids drawn at the 50% probability level. For clarity, disordered atoms are represented in a different style and the H atoms on disordered carbon atoms have been omitted. Dashed lines indicate hydrogen bonds.

The title compound consists of a binuclear Cu^II^ complex cation, a perchlorate anion and half of a solvent water mol­ecule (Fig. 1 ▸). Two copper(II) ions are bridged by a *cis-*oxamido group. The Cu1 atom, located at the inner site of the oxamide ligand, has a distorted square-planar geometry and is displaced from the coordination plane by 0.0454 (15) Å, which is consistent with structures reported previously (Gao & Wang, 2010 ▸; Lu *et al.*, 2011 ▸). The two *exo-*oxygen atoms of the oxamide ligand and two nitro­gen atoms of the Phdo mol­ecule chelate the Cu2 atom, forming the basal coordination plane [the maximum deviation being 0.0384 (14) Å for N4], and a water mol­ecule (O4) occupies the apical position, completing a distorted square-pyramidal coordination geometry with a τ value of 0.06 (Addison *et al.*, 1984 ▸). The Cu—O distance of 2.213 (3) Å in the apical direction is longer than those in the basal plane by 0.261 (4) and 0.266 (4) Å (Table 1 ▸). The Cu2 atom is displaced by 0.1610 (15) Å from the basal plane towards the apex.

The hexa­dentate oxamide anion, Dmapox^3−^, bridges the two copper(II) cations with three planar five-membered chelate rings and one six-membered ring, the latter being disordered over two positions. The puckering parameters of the first component (containing atoms C10*A* and C11*A*) are *Q* = 0.554 (8) Å, θ = 47.6 (6)° and ϕ = 206.0 (7)°, and those of the other are *Q* = 0.565 (11) Å, θ = 123.4 (8)° and ϕ = 38.8 (9)°; both suggest an approximate half-chair conformation.

## Supra­molecular features   

**Figure 2:**
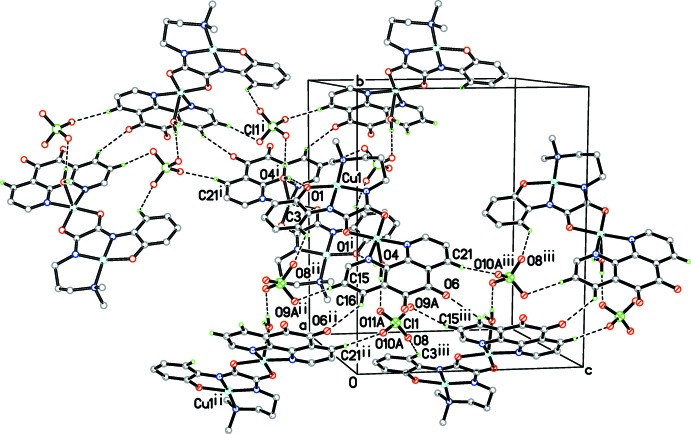
The two-dimensional wave-like hydrogen-bonding network constructed by classical O—H⋯O and weak C—H⋯O inter­actions [symmetry codes: (i) 1 − *x*, 1 − *y*, −*z*; (ii) *x*, 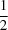 − *y*, *z* − 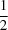; (iii) *x*, 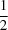 − *y*, *z* + 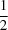].

**Figure 3:**
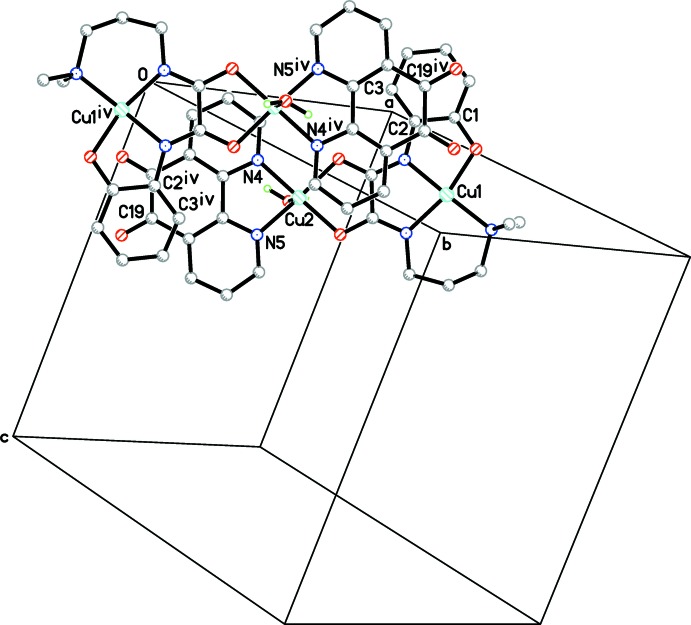
A perspective view of the π–π stacking inter­actions viewed perpendicular to the middle ring of the Phdo ligand. H atoms have been omitted for clarity [symmetry code: (iv) −*x*, 1 − *y*, −*z*].

Besides classical O—H⋯O hydrogen bonds, weak C—H⋯O hydrogen bonds and aromatic stacking inter­actions are important to the supra­molecular structure. As illustrated in Fig. 2 ▸, two symmetry-related binuclear cations link each other, forming a dimer by hydrogen bonds between the coordinating water mol­ecules and phenolic oxygen atoms (Table 2 ▸). Then the dimers are assembled by perchlorate anions, generating a wave-like layer parallel to (100). Subsequently, an offset π–π stacking inter­action occurs between the middle aromatic ring of the Phdo ligand of a binuclear unit and the benzene ring of the other unit at −*x*, 1 − *y*, −*z* [symmetry code (iv)], and *vice versa* (Fig. 3 ▸). The separations of the overlapped atoms from their opposite rings are 3.191 (4) (C2^iv^), 3.211 (4) (C3^iv^) and 3.252 (4) Å (C19^iv^).

## Database survey   

Several Cu^II^ complexes of 1,10-phenanthroline-5,6-dione have been reported previously, for example, Chetana *et al.* (2009 ▸); Galet *et al.* (2005 ▸); Saravani *et al.* (2007 ▸); Wang *et al.* (2013 ▸); Yamada *et al.* (2002 ▸) and Xu *et al.* (2006 ▸).

## Synthesis and crystallization   

*N*-[3-(Di­methyl­amino)­prop­yl]-*N*′-2-(oxidophen­yl)oxamide (H_3_Dmapox; Zhang *et al.*, 2013 ▸) and 1,10-phenanthroline-5,6-dione (Phdo; Dickeson & Summers, 1970 ▸) were prepared by published procedures. The title compound was obtained as follows: A solution of Cu(ClO_4_)_2_·6H_2_O (0.0371 g, 0.1 mmol) in methanol (5 ml) was added dropwise to a solution of H_3_Dmapox (0.0133 g, 0.05 mmol) and piperidine (0.0128 g, 0.15 mmol) in methanol (5 ml). The solution was stirred continuously for 0.5 h. Then a solution of Phdo (0.011 g, 0.05 mmol) in methanol (5 ml) was added dropwise, and the mixture was stirred continuously at 313 K for 6 h and then filtered. Dark-blue crystals of the title compound suitable for X-ray analysis were obtained from the filtrate by slow evaporation at room temperature for 7 d. Yield: 0.026 g (71.62%). Analysis calculated for Cu_2_C_25_H_25_ClN_5_O_10.5_: C 41.44, H 3.48, N 9.67%; found: C 42.57, H 3.15, N 9.19%.

## Refinement   

Crystal data, data collection, and refinement details are summarized in Table 3 ▸. Disorder occurs for four carbon atoms of the 3-(di­methyl­amino)­propyl group [C10*A*–C13*A*, with occupancies of 0.561 (11); C10*B*–C13*B*, 0.439 (11)], three oxygen atoms of the perchlorate ion [O9*A*–O11*A*, 0.646 (8); O9*B–*O11*B*, 0.354 (8)] and the solvent water mol­ecule (O7*A*, 0.207 (10); O7*B*, 0.293 (10)]. The occupancies were refined freely except for the sum of atoms O7*A* and O7*B* which was fixed at 0.5. Some restraints on distances (DFIX) and anisotropic displacement parameters (SIMU) were applied to the disordered atoms to avoid unreasonable geometries. The hydrogen atoms of the water mol­ecules were found in a difference Fourier map and then refined as riding. Other H atoms were placed in calculated positions, with C—H = 0.96 (meth­yl), 0.97 (methyl­ene) and 0.93 Å (aromatic), and refined using a riding model, with *U*_iso_(H) = 1.2 *U*_eq_(C) or 1.5 for methyl groups.

## Supplementary Material

Crystal structure: contains datablock(s) I, global. DOI: 10.1107/S2056989015009391/xu5850sup1.cif

Structure factors: contains datablock(s) I. DOI: 10.1107/S2056989015009391/xu5850Isup2.hkl

CCDC reference: 1401557

Additional supporting information:  crystallographic information; 3D view; checkCIF report

## Figures and Tables

**Table 1:** Selected bond lengths ()

Cu1O1	1.950(3)	Cu2O3	1.947(3)
Cu1N1	1.932(3)	Cu2O4	2.213(3)
Cu1N2	1.982(3)	Cu2N4	1.989(3)
Cu1N3	2.013(3)	Cu2N5	1.991(3)
Cu2O2	1.952(3)		

**Table 2:** Hydrogen-bond geometry (, )

*D*H*A*	*D*H	H*A*	*D* *A*	*D*H*A*
O4H4*A*O1^i^	0.84	1.89	2.666(3)	153
O4H4*B*O10*B*	0.91	1.93	2.740(8)	146
O4H4*B*O11*A*	0.91	2.10	3.009(7)	171
O7*A*H7*A*O3	0.86	2.51	3.27(2)	147
O7*B*H7*D*O5^ii^	0.86	2.51	3.305(18)	153
C3H3O8^iii^	0.93	2.52	3.193(5)	130
C15H15O9*A* ^iii^	0.93	2.55	3.335(7)	142
C15H15O11*B* ^iii^	0.93	2.49	3.384(10)	162
C16H16O6^iii^	0.93	2.54	3.195(5)	128
C10*A*H10*A*O6^ii^	0.97	2.44	3.202(9)	135
C11*A*H11*A*O4^iv^	0.97	2.54	3.409(7)	150
C13*A*H13*B*O4^i^	0.96	2.49	3.382(16)	154
C21H21O10*A* ^v^	0.93	2.53	3.277(8)	138
C23H23O7*A*	0.93	2.30	3.154(19)	152
C23H23O7*B*	0.93	2.49	3.31(2)	147

Symmetry codes: (i) 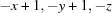; (ii) 
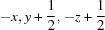
; (iii) 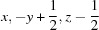; (iv) 
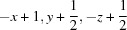
; (v) 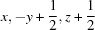.

**Table 3:** Experimental details

Crystal data	
Chemical formula	[Cu_2_(C_13_H_16_N_3_O_3_)(C_12_H_6_N_2_O_2_)(H_2_O)]ClO_4_0.5H_2_O
*M*_r_	726.03
Crystal system, space group	Monoclinic, *P*2_1_/*c*
Temperature (K)	296
*a*, *b*, *c* ()	11.7430(6), 17.3066(9), 14.1086(8)
()	98.154(1)
*V* (^3^)	2838.3(3)
*Z*	4
Radiation type	Mo *K*
(mm^1^)	1.66
Crystal size (mm)	0.30 0.12 0.06

Data collection	
Diffractometer	Bruker APEX area detector
Absorption correction	Multi-scan (*SADABS*; Bruker, 2002 ▸)
*T*_min_, *T*_max_	0.636, 0.907
No. of measured, independent and observed [*I* > 2(*I*)] reflections	21066, 6432, 4831
*R*_int_	0.054
(sin /)_max_ (^1^)	0.649

Refinement	
*R*[*F* ^2^ > 2(*F* ^2^)], *wR*(*F* ^2^), *S*	0.050, 0.114, 1.05
No. of reflections	6432
No. of parameters	478
No. of restraints	31
H-atom treatment	H-atom parameters constrained
_max_, _min_ (e ^3^)	0.76, 0.48

Computer programs: *SMART* and *SAINT* (Bruker, 2002 ▸), *SHELXS97*, *SHELXL97* and *XP* in *SHELXTL* (Sheldrick, 2008 ▸) and *WinGX* (Farrugia, 2012 ▸).

## supplementary crystallographic information

### Crystal data

**Table:**

[Cu_2_(C_13_H_16_N_3_O_3_)(C_12_H_6_N_2_O_2_)(H_2_O)]ClO_4_·0.5H_2_O	*F*(000) = 1476
*M**_r_* = 726.03	*D*_x_ = 1.699 Mg m^−^^3^
Monoclinic, *P*2_1_/*c*	Mo *K*α radiation, λ = 0.71073 Å
*a* = 11.7430 (6) Å	Cell parameters from 9065 reflections
*b* = 17.3066 (9) Å	θ = 3.2–27.4°
*c* = 14.1086 (8) Å	µ = 1.66 mm^−^^1^
β = 98.154 (1)°	*T* = 296 K
*V* = 2838.3 (3) Å^3^	Prism, dark blue
*Z* = 4	0.30 × 0.12 × 0.06 mm

### Data collection

**Table:**

Bruker APEX area-detector diffractometer	6432 independent reflections
Radiation source: fine-focus sealed tube	4831 reflections with *I* > 2σ(*I*)
Graphite monochromator	*R*_int_ = 0.054
φ and ω scans	θ_max_ = 27.5°, θ_min_ = 3.2°
Absorption correction: multi-scan (*SADABS*; Bruker, 2002)	*h* = −14→15
*T*_min_ = 0.636, *T*_max_ = 0.907	*k* = −22→22
21066 measured reflections	*l* = −15→18

### Refinement

**Table:**

Refinement on *F*^2^	Primary atom site location: structure-invariant direct methods
Least-squares matrix: full	Secondary atom site location: difference Fourier map
*R*[*F*^2^ > 2σ(*F*^2^)] = 0.050	Hydrogen site location: inferred from neighbouring sites
*wR*(*F*^2^) = 0.114	H-atom parameters constrained
*S* = 1.05	*w* = 1/[σ^2^(*F*_o_^2^) + (0.0369*P*)^2^ + 7.4021*P*] where *P* = (*F*_o_^2^ + 2*F*_c_^2^)/3
6432 reflections	(Δ/σ)_max_ < 0.001
478 parameters	Δρ_max_ = 0.76 e Å^−^^3^
31 restraints	Δρ_min_ = −0.47 e Å^−^^3^

### Special details

**Table:**

Geometry. All s.u.'s (except the s.u. in the dihedral angle between two l.s. planes) are estimated using the full covariance matrix. The cell s.u.'s are taken into account individually in the estimation of s.u.'s in distances, angles and torsion angles; correlations between s.u.'s in cell parameters are only used when they are defined by crystal symmetry. An approximate (isotropic) treatment of cell s.u.'s is used for estimating s.u.'s involving l.s. planes.
Refinement. Refinement of *F*^2^ against ALL reflections. The weighted *R*-factor *wR* and goodness of fit *S* are based on *F*^2^, conventional *R*-factors *R* are based on *F*, with *F* set to zero for negative *F*^2^. The threshold expression of *F*^2^ > 2σ(*F*^2^) is used only for calculating *R*-factors(gt) *etc*. and is not relevant to the choice of reflections for refinement. *R*-factors based on *F*^2^ are statistically about twice as large as those based on *F*, and *R*- factors based on ALL data will be even larger.

### Fractional atomic coordinates and isotropic or equivalent isotropic displacement parameters (Å^2^)

**Table:**

	*x*	*y*	*z*	*U*_iso_*/*U*_eq_	Occ. (<1)
Cu1	0.48982 (4)	0.62884 (2)	0.02261 (3)	0.01855 (12)	
Cu2	0.15568 (4)	0.46352 (3)	0.12248 (3)	0.01949 (12)	
O1	0.5443 (2)	0.62076 (14)	−0.10126 (18)	0.0201 (5)	
O2	0.2204 (2)	0.48428 (14)	0.00502 (19)	0.0202 (5)	
O3	0.2675 (2)	0.53779 (14)	0.18328 (19)	0.0216 (6)	
O4	0.2568 (2)	0.35877 (15)	0.1685 (2)	0.0260 (6)	
H4A	0.3245	0.3523	0.1580	0.07 (2)*	
H4B	0.2148	0.3146	0.1550	0.052 (16)*	
O5	−0.3086 (2)	0.27160 (16)	0.1260 (2)	0.0326 (7)	
O6	−0.2449 (3)	0.30625 (18)	0.3165 (2)	0.0376 (8)	
N1	0.3763 (2)	0.55502 (16)	−0.0332 (2)	0.0174 (6)	
N2	0.4133 (3)	0.61345 (17)	0.1376 (2)	0.0191 (6)	
N3	0.5927 (3)	0.71698 (18)	0.0732 (2)	0.0255 (7)	
N4	0.0213 (2)	0.40632 (17)	0.0548 (2)	0.0176 (6)	
N5	0.0705 (3)	0.45017 (19)	0.2338 (2)	0.0254 (7)	
C1	0.4728 (3)	0.5781 (2)	−0.1637 (3)	0.0193 (8)	
C2	0.3778 (3)	0.54062 (19)	−0.1310 (3)	0.0176 (7)	
C3	0.2998 (3)	0.4979 (2)	−0.1929 (3)	0.0214 (8)	
H3	0.2375	0.4744	−0.1706	0.026*	
C4	0.3151 (4)	0.4902 (2)	−0.2881 (3)	0.0270 (9)	
H4	0.2625	0.4623	−0.3303	0.032*	
C5	0.4095 (4)	0.5246 (2)	−0.3199 (3)	0.0286 (9)	
H5	0.4205	0.5186	−0.3835	0.034*	
C6	0.4876 (3)	0.5676 (2)	−0.2587 (3)	0.0249 (9)	
H6	0.5506	0.5897	−0.2815	0.030*	
C7	0.3069 (3)	0.52992 (19)	0.0233 (3)	0.0170 (7)	
C8	0.3321 (3)	0.56215 (19)	0.1238 (3)	0.0178 (7)	
C9	0.4368 (3)	0.6507 (2)	0.2314 (3)	0.0253 (9)	
H9A	0.4864	0.6178	0.2751	0.030*	0.561 (11)
H9B	0.3653	0.6579	0.2573	0.030*	0.561 (11)
H9C	0.3716	0.6824	0.2419	0.030*	0.439 (11)
H9D	0.4476	0.6116	0.2811	0.030*	0.439 (11)
C10A	0.4950 (7)	0.7291 (5)	0.2231 (6)	0.0207 (19)	0.561 (11)
H10A	0.4427	0.7629	0.1829	0.025*	0.561 (11)
H10B	0.5105	0.7525	0.2861	0.025*	0.561 (11)
C11A	0.6069 (6)	0.7230 (5)	0.1811 (4)	0.0213 (19)	0.561 (11)
H11A	0.6536	0.7680	0.2007	0.026*	0.561 (11)
H11B	0.6485	0.6778	0.2082	0.026*	0.561 (11)
C12A	0.5468 (14)	0.7891 (6)	0.0286 (10)	0.046 (4)	0.561 (11)
H12A	0.5380	0.7839	−0.0398	0.069*	0.561 (11)
H12B	0.5990	0.8306	0.0483	0.069*	0.561 (11)
H12C	0.4734	0.7999	0.0482	0.069*	0.561 (11)
C13A	0.7114 (8)	0.7022 (15)	0.0540 (12)	0.043 (5)	0.561 (11)
H13A	0.7615	0.7421	0.0830	0.065*	0.561 (11)
H13B	0.7128	0.7020	−0.0139	0.065*	0.561 (11)
H13C	0.7370	0.6530	0.0803	0.065*	0.561 (11)
C10B	0.5434 (12)	0.6994 (6)	0.2379 (7)	0.032 (3)	0.439 (11)
H10C	0.6093	0.6651	0.2436	0.038*	0.439 (11)
H10D	0.5493	0.7294	0.2965	0.038*	0.439 (11)
C11B	0.5519 (11)	0.7548 (5)	0.1554 (8)	0.036 (3)	0.439 (11)
H11C	0.4768	0.7773	0.1348	0.043*	0.439 (11)
H11D	0.6041	0.7965	0.1778	0.043*	0.439 (11)
C12B	0.5760 (13)	0.7781 (7)	−0.0015 (9)	0.038 (4)	0.439 (11)
H12D	0.6208	0.8228	0.0204	0.057*	0.439 (11)
H12E	0.4962	0.7920	−0.0139	0.057*	0.439 (11)
H12F	0.6005	0.7591	−0.0593	0.057*	0.439 (11)
C13B	0.7162 (10)	0.6988 (17)	0.0870 (15)	0.035 (5)	0.439 (11)
H13D	0.7595	0.7447	0.1058	0.053*	0.439 (11)
H13E	0.7375	0.6796	0.0281	0.053*	0.439 (11)
H13F	0.7323	0.6603	0.1360	0.053*	0.439 (11)
C14	0.0027 (3)	0.3843 (2)	−0.0364 (3)	0.0206 (8)	
H14	0.0530	0.4014	−0.0773	0.025*	
C15	−0.0881 (3)	0.3370 (2)	−0.0734 (3)	0.0231 (8)	
H15	−0.0984	0.3228	−0.1376	0.028*	
C16	−0.1632 (3)	0.3114 (2)	−0.0128 (3)	0.0227 (8)	
H16	−0.2245	0.2792	−0.0355	0.027*	
C17	−0.1456 (3)	0.3344 (2)	0.0822 (3)	0.0183 (7)	
C18	−0.2225 (3)	0.3083 (2)	0.1514 (3)	0.0234 (8)	
C19	−0.1890 (3)	0.3309 (2)	0.2573 (3)	0.0287 (9)	
C20	−0.0860 (3)	0.3801 (2)	0.2846 (3)	0.0251 (8)	
C21	−0.0471 (4)	0.4002 (3)	0.3791 (3)	0.0375 (11)	
H21	−0.0859	0.3833	0.4283	0.045*	
C22	0.0503 (4)	0.4458 (3)	0.3987 (3)	0.0455 (13)	
H22	0.0783	0.4595	0.4614	0.055*	
C23	0.1057 (4)	0.4706 (3)	0.3236 (3)	0.0387 (11)	
H23	0.1695	0.5027	0.3368	0.046*	
C24	−0.0232 (3)	0.4052 (2)	0.2139 (3)	0.0210 (8)	
C25	−0.0521 (3)	0.3817 (2)	0.1138 (3)	0.0170 (7)	
Cl1	0.04798 (9)	0.17375 (5)	0.17883 (7)	0.0275 (2)	
O8	0.0854 (3)	0.1132 (2)	0.2436 (3)	0.0567 (10)	
O9A	0.0082 (6)	0.2341 (3)	0.2331 (4)	0.054 (2)	0.646 (8)
O10A	−0.0380 (7)	0.1519 (4)	0.1034 (4)	0.060 (2)	0.646 (8)
O11A	0.1412 (6)	0.2040 (4)	0.1353 (5)	0.073 (2)	0.646 (8)
O9B	0.0505 (16)	0.1419 (9)	0.0878 (5)	0.112 (6)	0.354 (8)
O10B	0.1088 (10)	0.2417 (4)	0.1984 (12)	0.094 (6)	0.354 (8)
O11B	−0.0679 (5)	0.1910 (5)	0.1922 (7)	0.037 (3)	0.354 (8)
O7A	0.3514 (15)	0.5296 (11)	0.4141 (15)	0.056 (7)	0.207 (10)
H7A	0.3589	0.5251	0.3546	0.084*	0.207 (10)
H7B	0.4122	0.5110	0.4473	0.084*	0.207 (10)
O7B	0.282 (2)	0.5932 (11)	0.4515 (15)	0.146 (13)	0.293 (10)
H7C	0.2845	0.5848	0.5119	0.219*	0.293 (10)
H7D	0.2685	0.6416	0.4416	0.219*	0.293 (10)

### Atomic displacement parameters (Å^2^)

**Table:**

	*U*^11^	*U*^22^	*U*^33^	*U*^12^	*U*^13^	*U*^23^
Cu1	0.0132 (2)	0.0162 (2)	0.0274 (3)	−0.00272 (17)	0.00678 (18)	−0.00401 (18)
Cu2	0.0134 (2)	0.0205 (2)	0.0258 (3)	−0.00197 (18)	0.00698 (18)	−0.00077 (19)
O1	0.0139 (12)	0.0220 (13)	0.0252 (14)	−0.0045 (10)	0.0057 (11)	−0.0014 (11)
O2	0.0151 (12)	0.0197 (12)	0.0263 (14)	−0.0049 (10)	0.0053 (11)	−0.0003 (11)
O3	0.0186 (13)	0.0209 (12)	0.0270 (15)	−0.0024 (11)	0.0091 (11)	−0.0006 (11)
O4	0.0175 (14)	0.0236 (14)	0.0387 (17)	0.0015 (11)	0.0103 (12)	0.0083 (12)
O5	0.0260 (16)	0.0291 (15)	0.0463 (19)	−0.0087 (13)	0.0173 (14)	0.0000 (14)
O6	0.0327 (17)	0.0443 (18)	0.0399 (18)	−0.0003 (14)	0.0185 (15)	0.0153 (15)
N1	0.0135 (14)	0.0147 (14)	0.0245 (17)	0.0001 (12)	0.0041 (13)	−0.0010 (12)
N2	0.0159 (15)	0.0180 (15)	0.0235 (17)	0.0007 (12)	0.0028 (13)	−0.0013 (13)
N3	0.0198 (17)	0.0250 (16)	0.0334 (19)	−0.0058 (14)	0.0091 (15)	−0.0092 (15)
N4	0.0117 (14)	0.0198 (15)	0.0220 (17)	0.0017 (12)	0.0046 (12)	0.0032 (13)
N5	0.0196 (16)	0.0308 (17)	0.0268 (19)	0.0016 (14)	0.0061 (14)	−0.0042 (15)
C1	0.0145 (17)	0.0147 (16)	0.029 (2)	0.0046 (14)	0.0047 (15)	0.0007 (15)
C2	0.0123 (16)	0.0141 (16)	0.026 (2)	0.0015 (14)	0.0026 (15)	−0.0002 (15)
C3	0.0178 (18)	0.0181 (17)	0.028 (2)	−0.0005 (15)	0.0038 (16)	−0.0008 (15)
C4	0.024 (2)	0.0250 (19)	0.031 (2)	−0.0017 (17)	0.0011 (17)	−0.0070 (17)
C5	0.030 (2)	0.030 (2)	0.028 (2)	−0.0003 (18)	0.0109 (18)	−0.0026 (18)
C6	0.0203 (19)	0.0229 (18)	0.033 (2)	0.0003 (16)	0.0096 (17)	0.0025 (17)
C7	0.0112 (16)	0.0127 (15)	0.027 (2)	0.0016 (13)	0.0026 (14)	0.0017 (14)
C8	0.0140 (17)	0.0151 (16)	0.025 (2)	0.0023 (14)	0.0053 (15)	0.0000 (14)
C9	0.024 (2)	0.0260 (19)	0.027 (2)	−0.0045 (16)	0.0062 (17)	−0.0065 (16)
C10A	0.020 (4)	0.020 (4)	0.023 (4)	−0.002 (3)	0.005 (3)	−0.010 (3)
C11A	0.018 (4)	0.018 (4)	0.030 (4)	0.000 (3)	0.007 (3)	−0.009 (3)
C12A	0.067 (10)	0.018 (5)	0.050 (7)	−0.001 (5)	0.001 (6)	0.000 (5)
C13A	0.021 (5)	0.060 (8)	0.052 (11)	−0.024 (5)	0.013 (5)	−0.036 (9)
C10B	0.033 (7)	0.031 (6)	0.031 (6)	−0.002 (5)	0.004 (5)	−0.006 (5)
C11B	0.035 (7)	0.014 (5)	0.063 (8)	−0.002 (5)	0.019 (6)	−0.016 (5)
C12B	0.019 (7)	0.012 (5)	0.075 (12)	0.002 (4)	−0.022 (7)	−0.015 (6)
C13B	0.028 (6)	0.036 (6)	0.039 (12)	0.001 (5)	−0.006 (5)	−0.017 (9)
C14	0.0168 (18)	0.0226 (18)	0.024 (2)	0.0008 (15)	0.0068 (15)	0.0026 (15)
C15	0.023 (2)	0.0248 (19)	0.022 (2)	0.0029 (16)	0.0036 (16)	0.0012 (16)
C16	0.0124 (17)	0.0209 (18)	0.034 (2)	0.0026 (15)	0.0019 (16)	0.0039 (16)
C17	0.0129 (17)	0.0174 (16)	0.025 (2)	0.0002 (14)	0.0043 (15)	0.0030 (15)
C18	0.0176 (19)	0.0184 (17)	0.037 (2)	0.0048 (15)	0.0111 (17)	0.0071 (16)
C19	0.025 (2)	0.030 (2)	0.034 (2)	0.0055 (17)	0.0153 (18)	0.0113 (18)
C20	0.0212 (19)	0.029 (2)	0.026 (2)	0.0060 (17)	0.0095 (16)	0.0049 (17)
C21	0.031 (2)	0.059 (3)	0.025 (2)	0.008 (2)	0.0125 (19)	0.002 (2)
C22	0.032 (3)	0.079 (4)	0.026 (2)	−0.001 (3)	0.008 (2)	−0.013 (2)
C23	0.027 (2)	0.058 (3)	0.032 (3)	−0.004 (2)	0.0057 (19)	−0.013 (2)
C24	0.0140 (17)	0.0241 (18)	0.026 (2)	0.0037 (15)	0.0046 (15)	0.0021 (16)
C25	0.0116 (16)	0.0181 (17)	0.0220 (19)	0.0047 (14)	0.0049 (14)	0.0021 (14)
Cl1	0.0283 (5)	0.0253 (5)	0.0290 (5)	0.0042 (4)	0.0048 (4)	0.0013 (4)
O8	0.050 (2)	0.053 (2)	0.067 (3)	0.0233 (19)	0.0091 (19)	0.026 (2)
O9A	0.087 (6)	0.042 (3)	0.035 (3)	0.027 (4)	0.018 (3)	−0.001 (3)
O10A	0.075 (5)	0.063 (4)	0.037 (4)	−0.009 (4)	−0.015 (3)	0.002 (3)
O11A	0.089 (5)	0.066 (4)	0.076 (5)	−0.040 (4)	0.056 (4)	−0.018 (4)
O9B	0.111 (13)	0.175 (15)	0.048 (8)	0.063 (11)	0.006 (8)	−0.056 (9)
O10B	0.049 (7)	0.038 (6)	0.196 (16)	−0.019 (6)	0.024 (9)	0.008 (9)
O11B	0.016 (4)	0.043 (6)	0.051 (6)	0.009 (4)	0.001 (4)	0.016 (5)
O7A	0.043 (11)	0.056 (13)	0.073 (14)	0.001 (9)	0.022 (9)	0.000 (10)
O7B	0.27 (3)	0.065 (13)	0.096 (16)	−0.036 (17)	−0.008 (18)	0.009 (11)

### Geometric parameters (Å, º)

**Table:**

Cu1—O1	1.950 (3)	C10A—H10B	0.9700
Cu1—N1	1.932 (3)	C11A—H11A	0.9700
Cu1—N2	1.982 (3)	C11A—H11B	0.9700
Cu1—N3	2.013 (3)	C12A—H12A	0.9600
Cu2—O2	1.952 (3)	C12A—H12B	0.9600
Cu2—O3	1.947 (3)	C12A—H12C	0.9600
Cu2—O4	2.213 (3)	C13A—H13A	0.9600
Cu2—N4	1.989 (3)	C13A—H13B	0.9600
Cu2—N5	1.991 (3)	C13A—H13C	0.9600
O1—C1	1.347 (4)	C10B—C11B	1.522 (9)
O2—C7	1.284 (4)	C10B—H10C	0.9700
O3—C8	1.280 (4)	C10B—H10D	0.9700
O4—H4A	0.8374	C11B—H11C	0.9700
O4—H4B	0.9146	C11B—H11D	0.9700
O5—C18	1.205 (5)	C12B—H12D	0.9600
O6—C19	1.211 (5)	C12B—H12E	0.9600
N1—C7	1.293 (4)	C12B—H12F	0.9600
N1—C2	1.405 (5)	C13B—H13D	0.9600
N2—C8	1.297 (4)	C13B—H13E	0.9600
N2—C9	1.463 (5)	C13B—H13F	0.9600
N3—C12A	1.465 (8)	C14—C15	1.385 (5)
N3—C13B	1.470 (9)	C14—H14	0.9300
N3—C11B	1.470 (10)	C15—C16	1.386 (5)
N3—C13A	1.480 (8)	C15—H15	0.9300
N3—C12B	1.487 (9)	C16—C17	1.386 (5)
N3—C11A	1.511 (6)	C16—H16	0.9300
N4—C14	1.331 (5)	C17—C25	1.390 (5)
N4—C25	1.349 (4)	C17—C18	1.491 (5)
N5—C23	1.324 (5)	C18—C19	1.541 (6)
N5—C24	1.345 (5)	C19—C20	1.484 (6)
C1—C6	1.388 (5)	C20—C21	1.391 (6)
C1—C2	1.423 (5)	C20—C24	1.391 (5)
C2—C3	1.386 (5)	C21—C22	1.384 (7)
C3—C4	1.387 (6)	C21—H21	0.9300
C3—H3	0.9300	C22—C23	1.388 (6)
C4—C5	1.388 (5)	C22—H22	0.9300
C4—H4	0.9300	C23—H23	0.9300
C5—C6	1.385 (6)	C24—C25	1.463 (5)
C5—H5	0.9300	Cl1—O10B	1.383 (5)
C6—H6	0.9300	Cl1—O9B	1.401 (5)
C7—C8	1.513 (5)	Cl1—O10A	1.411 (6)
C9—C10B	1.501 (11)	Cl1—O9A	1.413 (5)
C9—C10A	1.532 (8)	Cl1—O8	1.419 (3)
C9—H9A	0.9700	Cl1—O11A	1.429 (4)
C9—H9B	0.9700	Cl1—O11B	1.432 (5)
C9—H9C	0.9700	O7A—H7A	0.8596
C9—H9D	0.9700	O7A—H7B	0.8602
C10A—C11A	1.519 (7)	O7B—H7C	0.8602
C10A—H10A	0.9700	O7B—H7D	0.8603
			
O1—Cu1—N1	83.25 (11)	H10A—C10A—H10B	107.8
O1—Cu1—N2	165.77 (11)	N3—C11A—C10A	114.8 (6)
O1—Cu1—N3	96.65 (11)	N3—C11A—H11A	108.6
N1—Cu1—N2	83.00 (13)	C10A—C11A—H11A	108.6
N1—Cu1—N3	172.12 (13)	N3—C11A—H11B	108.6
N2—Cu1—N3	97.48 (13)	C10A—C11A—H11B	108.6
O2—Cu2—O3	86.16 (10)	H11A—C11A—H11B	107.6
O2—Cu2—N4	93.58 (11)	N3—C12A—H12A	109.5
O2—Cu2—N5	172.09 (12)	N3—C12A—H12B	109.5
O3—Cu2—N4	168.40 (11)	N3—C12A—H12C	109.5
O3—Cu2—N5	96.42 (12)	N3—C13A—H13A	109.5
N4—Cu2—N5	82.38 (13)	N3—C13A—H13B	109.5
O4—Cu2—O2	98.20 (10)	N3—C13A—H13C	109.5
O4—Cu2—O3	96.39 (11)	C9—C10B—C11B	116.9 (9)
O4—Cu2—N4	95.12 (11)	C9—C10B—H10C	108.1
O4—Cu2—N5	88.97 (12)	C11B—C10B—H10C	108.1
C1—O1—Cu1	111.8 (2)	C9—C10B—H10D	108.1
C7—O2—Cu2	109.6 (2)	C11B—C10B—H10D	108.1
C8—O3—Cu2	110.7 (2)	H10C—C10B—H10D	107.3
Cu2—O4—H4A	122.8	N3—C11B—C10B	112.7 (9)
Cu2—O4—H4B	111.8	N3—C11B—H11C	109.0
H4A—O4—H4B	110.4	C10B—C11B—H11C	109.0
C7—N1—C2	130.0 (3)	N3—C11B—H11D	109.0
C7—N1—Cu1	115.4 (3)	C10B—C11B—H11D	109.0
C2—N1—Cu1	114.4 (2)	H11C—C11B—H11D	107.8
C8—N2—C9	118.6 (3)	N3—C12B—H12D	109.5
C8—N2—Cu1	112.2 (2)	N3—C12B—H12E	109.5
C9—N2—Cu1	129.2 (2)	H12D—C12B—H12E	109.5
C13B—N3—C11B	114.7 (10)	N3—C12B—H12F	109.5
C12A—N3—C13A	111.5 (11)	H12D—C12B—H12F	109.5
C13B—N3—C12B	105.8 (12)	H12E—C12B—H12F	109.5
C11B—N3—C12B	102.6 (7)	N3—C13B—H13D	109.5
C12A—N3—C11A	110.8 (6)	N3—C13B—H13E	109.5
C13A—N3—C11A	103.0 (7)	H13D—C13B—H13E	109.5
C12A—N3—Cu1	109.1 (7)	N3—C13B—H13F	109.5
C13B—N3—Cu1	114.6 (12)	H13D—C13B—H13F	109.5
C11B—N3—Cu1	111.8 (4)	H13E—C13B—H13F	109.5
C13A—N3—Cu1	109.7 (9)	N4—C14—C15	123.0 (3)
C12B—N3—Cu1	106.0 (6)	N4—C14—H14	118.5
C11A—N3—Cu1	112.6 (3)	C15—C14—H14	118.5
C14—N4—C25	118.3 (3)	C14—C15—C16	118.7 (4)
C14—N4—Cu2	128.1 (2)	C14—C15—H15	120.6
C25—N4—Cu2	113.2 (2)	C16—C15—H15	120.6
C23—N5—C24	119.3 (4)	C17—C16—C15	118.9 (4)
C23—N5—Cu2	126.9 (3)	C17—C16—H16	120.6
C24—N5—Cu2	113.0 (3)	C15—C16—H16	120.6
O1—C1—C6	123.5 (3)	C16—C17—C25	118.8 (3)
O1—C1—C2	118.8 (3)	C16—C17—C18	121.7 (3)
C6—C1—C2	117.6 (3)	C25—C17—C18	119.5 (3)
C3—C2—N1	127.9 (3)	O5—C18—C17	121.8 (4)
C3—C2—C1	121.2 (3)	O5—C18—C19	120.6 (3)
N1—C2—C1	110.9 (3)	C17—C18—C19	117.6 (3)
C2—C3—C4	119.8 (3)	O6—C19—C20	121.7 (4)
C2—C3—H3	120.1	O6—C19—C18	119.4 (4)
C4—C3—H3	120.1	C20—C19—C18	118.9 (3)
C3—C4—C5	119.4 (4)	C21—C20—C24	118.3 (4)
C3—C4—H4	120.3	C21—C20—C19	122.5 (4)
C5—C4—H4	120.3	C24—C20—C19	119.2 (4)
C6—C5—C4	121.2 (4)	C22—C21—C20	118.9 (4)
C6—C5—H5	119.4	C22—C21—H21	120.5
C4—C5—H5	119.4	C20—C21—H21	120.5
C5—C6—C1	120.7 (4)	C21—C22—C23	119.2 (4)
C5—C6—H6	119.7	C21—C22—H22	120.4
C1—C6—H6	119.7	C23—C22—H22	120.4
O2—C7—N1	129.3 (4)	N5—C23—C22	122.1 (4)
O2—C7—C8	117.3 (3)	N5—C23—H23	119.0
N1—C7—C8	113.4 (3)	C22—C23—H23	119.0
O3—C8—N2	128.1 (3)	N5—C24—C20	122.1 (4)
O3—C8—C7	116.0 (3)	N5—C24—C25	115.7 (3)
N2—C8—C7	115.8 (3)	C20—C24—C25	122.1 (3)
N2—C9—C10B	110.6 (5)	N4—C25—C17	122.2 (3)
N2—C9—C10A	110.5 (4)	N4—C25—C24	115.2 (3)
N2—C9—H9A	109.6	C17—C25—C24	122.5 (3)
C10A—C9—H9A	109.6	O10B—Cl1—O9B	116.1 (10)
N2—C9—H9B	109.6	O10A—Cl1—O9A	110.6 (4)
C10A—C9—H9B	109.6	O10B—Cl1—O8	113.3 (6)
H9A—C9—H9B	108.1	O9B—Cl1—O8	104.8 (5)
N2—C9—H9C	109.6	O10A—Cl1—O8	114.2 (3)
C10B—C9—H9C	109.9	O9A—Cl1—O8	107.0 (3)
N2—C9—H9D	109.6	O10A—Cl1—O11A	106.5 (4)
C10B—C9—H9D	109.0	O9A—Cl1—O11A	107.2 (4)
H9C—C9—H9D	108.1	O8—Cl1—O11A	111.2 (4)
C11A—C10A—C9	112.8 (6)	O10B—Cl1—O11B	105.7 (7)
C11A—C10A—H10A	109.0	O9B—Cl1—O11B	110.3 (8)
C9—C10A—H10A	109.0	O8—Cl1—O11B	106.4 (4)
C11A—C10A—H10B	109.0	H7A—O7A—H7B	107.8
C9—C10A—H10B	109.0	H7C—O7B—H7D	107.8
			
N1—Cu1—O1—C1	7.0 (2)	Cu2—O3—C8—N2	177.6 (3)
N2—Cu1—O1—C1	22.0 (6)	Cu2—O3—C8—C7	0.8 (4)
N3—Cu1—O1—C1	−165.1 (2)	C9—N2—C8—O3	−0.8 (6)
O3—Cu2—O2—C7	4.6 (2)	Cu1—N2—C8—O3	178.8 (3)
N4—Cu2—O2—C7	173.0 (2)	C9—N2—C8—C7	176.0 (3)
O4—Cu2—O2—C7	−91.3 (2)	Cu1—N2—C8—C7	−4.4 (4)
O2—Cu2—O3—C8	−2.9 (2)	O2—C7—C8—O3	3.3 (5)
N4—Cu2—O3—C8	−92.0 (6)	N1—C7—C8—O3	−178.5 (3)
N5—Cu2—O3—C8	−175.4 (2)	O2—C7—C8—N2	−174.0 (3)
O4—Cu2—O3—C8	94.9 (2)	N1—C7—C8—N2	4.3 (4)
O1—Cu1—N1—C7	176.1 (3)	C8—N2—C9—C10B	172.0 (6)
N2—Cu1—N1—C7	−0.2 (3)	Cu1—N2—C9—C10B	−7.5 (7)
O1—Cu1—N1—C2	−7.7 (2)	C8—N2—C9—C10A	−156.4 (5)
N2—Cu1—N1—C2	176.0 (2)	Cu1—N2—C9—C10A	24.1 (6)
N1—Cu1—N2—C8	2.7 (2)	N2—C9—C10A—C11A	−58.1 (8)
O1—Cu1—N2—C8	−12.3 (6)	C12A—N3—C11A—C10A	67.3 (10)
N3—Cu1—N2—C8	174.8 (2)	C13A—N3—C11A—C10A	−173.3 (12)
N1—Cu1—N2—C9	−177.8 (3)	Cu1—N3—C11A—C10A	−55.2 (7)
O1—Cu1—N2—C9	167.2 (4)	C9—C10A—C11A—N3	80.9 (9)
N3—Cu1—N2—C9	−5.7 (3)	N2—C9—C10B—C11B	48.9 (12)
O1—Cu1—N3—C12A	76.8 (6)	C13B—N3—C11B—C10B	−74.4 (16)
N2—Cu1—N3—C12A	−105.0 (6)	C12B—N3—C11B—C10B	171.4 (11)
O1—Cu1—N3—C13B	−64.5 (10)	Cu1—N3—C11B—C10B	58.2 (11)
N2—Cu1—N3—C13B	113.8 (10)	C9—C10B—C11B—N3	−82.3 (14)
O1—Cu1—N3—C11B	162.9 (5)	C25—N4—C14—C15	−0.3 (5)
N2—Cu1—N3—C11B	−18.9 (6)	Cu2—N4—C14—C15	172.5 (3)
O1—Cu1—N3—C13A	−45.6 (9)	N4—C14—C15—C16	0.0 (6)
N2—Cu1—N3—C13A	132.6 (9)	C14—C15—C16—C17	0.6 (5)
O1—Cu1—N3—C12B	51.8 (6)	C15—C16—C17—C25	−0.8 (5)
N2—Cu1—N3—C12B	−129.9 (6)	C15—C16—C17—C18	−179.4 (3)
O1—Cu1—N3—C11A	−159.7 (4)	C16—C17—C18—O5	−6.4 (6)
N2—Cu1—N3—C11A	18.6 (4)	C25—C17—C18—O5	175.0 (3)
O3—Cu2—N4—C14	96.8 (6)	C16—C17—C18—C19	174.4 (3)
O2—Cu2—N4—C14	8.5 (3)	C25—C17—C18—C19	−4.2 (5)
N5—Cu2—N4—C14	−178.4 (3)	O5—C18—C19—O6	5.9 (6)
O4—Cu2—N4—C14	−90.1 (3)	C17—C18—C19—O6	−174.9 (3)
O3—Cu2—N4—C25	−90.1 (6)	O5—C18—C19—C20	−176.5 (3)
O2—Cu2—N4—C25	−178.5 (2)	C17—C18—C19—C20	2.7 (5)
N5—Cu2—N4—C25	−5.3 (2)	O6—C19—C20—C21	0.5 (6)
O4—Cu2—N4—C25	83.0 (2)	C18—C19—C20—C21	−177.1 (4)
O3—Cu2—N5—C23	−15.8 (4)	O6—C19—C20—C24	178.4 (4)
N4—Cu2—N5—C23	175.8 (4)	C18—C19—C20—C24	0.8 (5)
O4—Cu2—N5—C23	80.5 (4)	C24—C20—C21—C22	1.6 (6)
O3—Cu2—N5—C24	174.7 (3)	C19—C20—C21—C22	179.5 (4)
N4—Cu2—N5—C24	6.4 (3)	C20—C21—C22—C23	0.6 (7)
O4—Cu2—N5—C24	−88.9 (3)	C24—N5—C23—C22	1.4 (7)
Cu1—O1—C1—C6	175.6 (3)	Cu2—N5—C23—C22	−167.4 (4)
Cu1—O1—C1—C2	−5.4 (4)	C21—C22—C23—N5	−2.2 (8)
C7—N1—C2—C3	3.4 (6)	C23—N5—C24—C20	0.9 (6)
Cu1—N1—C2—C3	−172.1 (3)	Cu2—N5—C24—C20	171.2 (3)
C7—N1—C2—C1	−177.8 (3)	C23—N5—C24—C25	−176.6 (4)
Cu1—N1—C2—C1	6.6 (4)	Cu2—N5—C24—C25	−6.3 (4)
O1—C1—C2—C3	178.2 (3)	C21—C20—C24—N5	−2.4 (6)
C6—C1—C2—C3	−2.8 (5)	C19—C20—C24—N5	179.6 (3)
O1—C1—C2—N1	−0.7 (4)	C21—C20—C24—C25	175.0 (4)
C6—C1—C2—N1	178.3 (3)	C19—C20—C24—C25	−3.0 (5)
N1—C2—C3—C4	179.7 (3)	C14—N4—C25—C17	0.0 (5)
C1—C2—C3—C4	1.0 (5)	Cu2—N4—C25—C17	−173.8 (3)
C2—C3—C4—C5	1.0 (6)	C14—N4—C25—C24	177.2 (3)
C3—C4—C5—C6	−1.2 (6)	Cu2—N4—C25—C24	3.4 (4)
C4—C5—C6—C1	−0.6 (6)	C16—C17—C25—N4	0.5 (5)
O1—C1—C6—C5	−178.5 (3)	C18—C17—C25—N4	179.1 (3)
C2—C1—C6—C5	2.6 (5)	C16—C17—C25—C24	−176.4 (3)
Cu2—O2—C7—N1	176.7 (3)	C18—C17—C25—C24	2.2 (5)
Cu2—O2—C7—C8	−5.4 (4)	N5—C24—C25—N4	2.0 (5)
C2—N1—C7—O2	0.5 (6)	C20—C24—C25—N4	−175.6 (3)
Cu1—N1—C7—O2	176.1 (3)	N5—C24—C25—C17	179.2 (3)
C2—N1—C7—C8	−177.5 (3)	C20—C24—C25—C17	1.6 (5)
Cu1—N1—C7—C8	−1.9 (4)		

### Hydrogen-bond geometry (Å, º)

**Table:**

*D*—H···*A*	*D*—H	H···*A*	*D*···*A*	*D*—H···*A*
O4—H4*A*···O1^i^	0.84	1.89	2.666 (3)	153
O4—H4*B*···O10*B*	0.91	1.93	2.740 (8)	146
O4—H4*B*···O11*A*	0.91	2.10	3.009 (7)	171
O7*A*—H7*A*···O3	0.86	2.51	3.27 (2)	147
O7*B*—H7*D*···O5^ii^	0.86	2.51	3.305 (18)	153
C3—H3···O8^iii^	0.93	2.52	3.193 (5)	130
C15—H15···O9*A*^iii^	0.93	2.55	3.335 (7)	142
C15—H15···O11*B*^iii^	0.93	2.49	3.384 (10)	162
C16—H16···O6^iii^	0.93	2.54	3.195 (5)	128
C10*A*—H10*A*···O6^ii^	0.97	2.44	3.202 (9)	135
C11*A*—H11*A*···O4^iv^	0.97	2.54	3.409 (7)	150
C13*A*—H13*B*···O4^i^	0.96	2.49	3.382 (16)	154
C21—H21···O10*A*^v^	0.93	2.53	3.277 (8)	138
C23—H23···O7*A*	0.93	2.30	3.154 (19)	152
C23—H23···O7*B*	0.93	2.49	3.31 (2)	147

Symmetry codes: (i) −*x*+1, −*y*+1, −*z*; (ii) −*x*, *y*+1/2, −*z*+1/2; (iii) *x*, −*y*+1/2, *z*−1/2; (iv) −*x*+1, *y*+1/2, −*z*+1/2; (v) *x*, −*y*+1/2, *z*+1/2.
